# Hepatitis C virus incidence trend and its risk factors among people who inject drugs in Hai Phong, Vietnam

**DOI:** 10.1007/s12072-025-10856-w

**Published:** 2025-07-17

**Authors:** Hong Thi Tran, Huong Thi Duong, Khue Minh Pham, Binh Thanh Nguyen, Roselyne Vallo, Morgana D’Ottavi, Giang Thi Hoang, Vinh Hai Vu, Oanh Thi Hai Khuat, Thanh Tuyet Thi Nham, Duc Quang Nguyen, Catherine Quillet, Delphine Rapoud, Philippe Van de Perre, Jonathan Feelemyer, Laurent Michel, Didier Laureillard, Don Des Jarlais, Jean-Pierre Moles, Nicolas Nagot

**Affiliations:** 1 Department of Public Health, Hai Phong University of Medicine and Pharmacy, 72A Nguyen Binh Khiem Street, Ngo Quyen Dis, Hai Phong, Vietnam; 2 Pathogenesis and Control of Chronic and Emerging Infections, University of Montpellier, INSERM, Etablissement Francais du Sang, University of Antilles, 60 Rue de Navacelles, 34394 Montpellier, France; 3 Infectious and Tropical Diseases Department, Viet Tiep Hospital, 1 Nha Thuong Street, Le Chan Dis, Hai Phong, Hai Phong Vietnam; 4 Supporting Community Development Initiatives, No.9 165/30 Alley, Thai Ha Street, Lang Ha Ward, Dong Da District, Hanoi, Vietnam; 5 School of Global Public Health, New York University, 708 Broadway, New York, NY 10003 USA; 6 Paris Saclay University, Pierre Nicole Center, French Red Cross, CESP Inserm UMRS 1018, Paris, France; 7 Infectious Diseases Department, Caremeau University Hospital, Place du Professeur Robert Debre, 30029 Nımes, France

**Keywords:** HCV infection, PWID, HIV, Determinants, Active heroin injection, PLWH

## Abstract

**Background:**

HCV incidence among people who inject drugs (PWID) remains unacceptably high. Using the data from the DRIVE study, we aimed to describe HCV incidence trends and investigate its associated risk factors among PWID in Hai Phong, Vietnam.

**Methods:**

Active PWID were recruited through 3 annual respondent-driven sampling (RDS) surveys; part of them were included in the study cohorts. HCV seroincidence was calculated for PWID participating in multiple surveys (recaptures) or in cohorts. A nested case–control design was used for risk factor analysis. Controls were matched to HCV seroconversion cases on age, sex, cohort participation and HCV seroconversion visit. Risk factors were measured over the period preceding the HCV seroconversion visits.

**Results:**

There were 83 HCV seroconversions during 844 person-years in 540 included participants. The overall HCV incidence was 9.8/100 person-years (95% CI 7.9–12.2). HCV incidence decreased over follow-up time and was particularly high among PWID living with HIV (PLWH), i.e., 37.2/100 person-years (95% CI 26.4–52.3). HIV infection (OR = 10.0, 95% CI 6.8–16.2) and active heroin injection (OR: 3.2, 95% CI 2.3–4.8) were associated with a higher risk of HCV seroconversion for cohort participants. Among RDS recaptures, living with a sexual partner and currently using methadone had opposite effects on HCV incidence, OR = 2.9, 95% CI 2.2–4.3 and OR = 0.4, 95% CI 0.3–0.5, respectively.

**Conclusion:**

HCV incidence among PWID in Hai Phong was still 5 times higher than the WHO target for elimination. Along with strengthened HCV prevention programs, affordable HCV treatment should be made available for PWID to reach the elimination goal.

**Graphical abstract:**

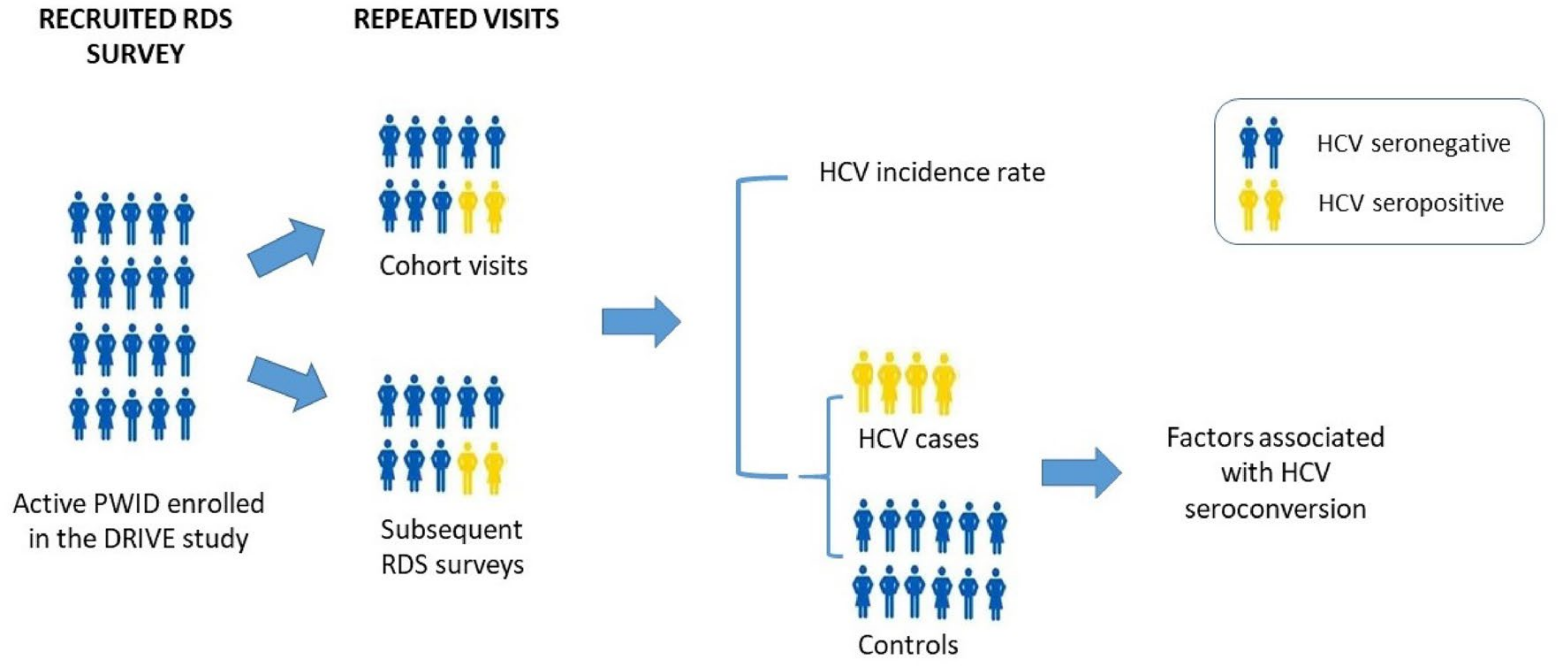

**Supplementary Information:**

The online version contains supplementary material available at 10.1007/s12072-025-10856-w.

## Introduction

Hepatitis C virus (HCV) infection is one of the major health problems, with around 50 million people infected worldwide in 2022 [1]. Chronic HCV infection can lead to serious complications such as cirrhosis and hepatocellular carcinoma, which accounted for the majority of 1.3 million deaths due to viral hepatitis. People who inject drugs (PWID), about 14.8 million worldwide, is the group having the highest burden of HCV infection with a prevalence of approximately 38.8%, which accounts for about 10% of people living with HCV worldwide in 2021 [2]. Transmission of HCV in this group is known to occur through unsafe injecting practices, although specific risky injecting practices vary from place to place. Globally, almost 44% of HCV incidence was estimated to occur among PWID [1]. This high dynamic of HCV transmission among PWID makes HCV control in this group play a pivotal role in the overall success of the World Health Organisation (WHO) HCV elimination program. Indeed, WHO has set a specific incidence target for this group of ≤ 2/100 PWID by 2030, which represents an 80% reduction from the 2015 baseline level [3].

Achieving this ambitious goal requires the high coverage of preventive interventions and large-scale test and treat programs [3]. Harm reduction interventions recommended by WHO for PWID include needle syringe program (NSP) and opioid substitution treatment (OST) [3]. However, NSP appeared to be less efficient in preventing HCV transmission than HIV transmission [4]. Therefore, optimization of preventive interventions is crucial to break the transmission chains and eliminate HCV as a public health threat.

In Vietnam, the seroprevalence of HCV infection in the general population was approximately 0.26%, but it was as high as 57.8% among PWID [5]. The difficulties in controlling HCV infection among PWID were highlighted in the DRIVE-IN study conducted in Hai Phong, Vietnam in 2014 [6]. The results showed that the HIV control programs, including NSP and OST, reduced HIV incidence (0/100 person-years, one-sided 97.5% CI 0–1.8), but not HCV incidence, i.e., 19.4/100 person-years (95% CI 11.5–30.7), although the two infections have similar routes of transmission. In addition, HCV treatment has not been scaled up, so the majority of PWID remain undiagnosed and untreated, and continue to spread the virus unknowingly. The above data showed that all aspects of the HCV cascade in Vietnam remain suboptimal and should be improved in order to reach the elimination target of WHO by 2030, especially among PWID.

Studies on HCV incidence and its specific risk factors among PWID in Vietnam are scarce and outdated as shown in a systematic review [7]. Therefore, we took advantage of the available data from the DRIVE study [8], the successive study of DRIVE-IN with a larger sample size and longer follow-up, to investigate more comprehensively the current dynamics of HCV transmission among PWID in Hai Phong. This is the third biggest city of Vietnam, with around 2 million inhabitants and an estimated population of 5000 people currently injecting drugs using capture–recapture methods at the beginning of the DRIVE study [9]. With good coverage of OST (42% of participants used methadone in our first intervention) and NSP as well as strong peer/community groups, Hai Phong was chosen to implement the community-based interventions to address multiple serious health problems of hard-to-reach populations [8]. In this study, specifically, we aimed at describing HCV incidence and investigating factors associated with the risk of HCV seroconversion including potential routes of HCV transmission among PWID during the DRIVE follow-up.

## Materials and methods

### Study design

This ancillary study used data from the interventional DRIVE (Drug use and infections in Vietnam: Ending the HIV epidemic among persons who inject drugs in Hai Phong, Viet Nam) project (NCT03526939). The DRIVE study evaluated a community-based interventions for the control of HIV transmission among PWID in Hai Phong, Vietnam [8]. It recruited PWID through three annual respondent-driven sampling (RDS) surveys implemented between 2016 and 2018, each including about 1400 PWID. In addition, a fourth RDS survey was carried out in 2019 for evaluation purpose. As multiple participation in different surveys was allowed, number of distinct individuals recruited was 3150 [10]. Each survey fueled 2 cohorts, one cohort including PLWH and the other including a corresponding number of HIV-negative PWID (Supplementary Fig. 1). After three RDS surveys, more than 800 PWID were included in each cohort. These participants were followed up every 6 months until the end of the study period, i.e., 2020. At either RDS surveys or cohort follow-up visits, information on socio-demographic characteristics, current drug and alcohol use and other risky behaviors were collected using standardized face-to-face questionnaires administered by trained community-based organization (CBO) members [8]. In addition, HIV and HCV rapid tests (SD Bioline, Standard Diagnostic, Inc, Korea) were performed for those whose status was not known or negative. All participants received counseling on HCV prevention, were provided harm reduction materials and supported for OST initiation. The DRIVE study was approved by the Institutional Review Board (IRB) of New York University and Hai Phong University of Medicine and Pharmacy, Vietnam.

We used a nested case–control study design to investigate the risk factors associated with HCV seroconversion. This secondary analysis of the DRIVE study was approved by the Ethics Committee of the London School of Hygiene and Tropical Medicine.

### Study population

Participants of the DRIVE study were active PWID, i.e., currently injecting drugs, confirmed by the detection of heroin in urine and presence of recent injection skin marks. In this work, participants with an HCV seronegative test at enrollment (first RDS survey participation) and having at least one follow-up visit (cohort follow-up visits or subsequent RDS surveys) were included in the analysis. For the risk factor analysis, cases were participants who had HCV seroconversion at any visits. Three controls were randomly selected to match each case on sex, age at baseline (5-year intervals), the index visit, i.e., the visit of HCV seroconversion, and cohort participation at the index visit.

### Data collection

For the risk factor analysis, socio-demographic and economic characteristics, i.e., education, living with a sexual partner, having a regular place to stay, recent monthly income, HIV status, were assessed. In addition, numerous risky behavior factors, i.e., active heroin injection, daily injection, injecting with used syringes/needles, sharing drugs with used syringes, using water/novocain used by others, smoking methamphetamine, using other non-injected drugs, at-risk alcohol consumption, having sex while taking methamphetamine, having new tattoo/piercing, helping others inject for the first time, being incarcerated overnight, currently using methadone, and overdose were analyzed to consolidate the factors associated with a higher risk of HCV seroconversion. Currently using methadone was based on urine test results of methadone when available; otherwise, participant responses in the questionnaires were used. At-risk alcohol consumption, assessed by the AUDIT-C questionnaire, was defined as a score ≥ 3 for females and ≥ 4 for males [11]. Data at baseline of socio-demographic and economic variables were used. Data on time varying factors were taken at the index visit, so they were more likely captured during the exposure period, i.e., the period during which HCV-transmitting contacts occurred.

### Statistical analysis

In order to estimate a potential selection bias, participant characteristics at baseline were described and compared to HCV seronegative participants who were not included in this analysis due to the absence of follow-up. We used Chi-squared test to compared categorical variables and t-test/Wilcoxon Rank Sum test for continuous variables. Overall HCV incidence was calculated with the number of HCV seroconverters divided by the total follow-up time of participants. For each participant, total follow-up time was calculated from the first RDS participation to the last cohort follow-up visits or to the last participation in RDS surveys for RDS recaptures. Participants with HCV seroconversion had their follow-up time censored at mid-point between the last visit before the index visit and the index visit. Results were stratified by age, HIV status and cohort participation. The trend of HCV incidence was assessed by comparing HCV incidence of participants with 6–12 months, 12–24 months or more than 24 months of follow-up, also stratified by HIV and cohort participation.

The risk factor analysis was performed separately for cohort participants and RDS recaptures as these two groups were different in terms of HIV status and active heroin injection. Both univariable and multivariable analyses were done by conditional logistic regression. Multicollinearity was checked before we performed multivariable analysis. We used the backward procedure to build the multivariable model, with the initial model including age and all variables having a p-value of univariable analysis of less than 0.2, excluding variables with high proportion of missing data. The full model and the models with one variable less were compared using the AIC and the likelihood ratio test (LRT) to detect and drop the variable with the lowest AIC and the highest p-value of LRT. With the best-fit approach, the process was repeated until only variables improving model fit were retained. HIV infection was also examined as a potential effect modifier in the final model. Every time the model was run, different controls were matched to cases. Therefore, we ran the model 10 times to check the impact of random selection of controls on the results of multivariable analysis and record the lists of distinct variables associated with HCV seroconversion out of these runs. Then, the final model was replicated 1000 times with changing in random selection of controls. After checking for normal distribution of the outputs, the mean ORs and their 95% CI of these replications were used. All statistical analyses were done using STATA/IC 16.1 (Stata Corp, College Station, Texas).

## Results

### Description of the study participants at baseline

Among 3150 distinct DRIVE participants, 1025 (32.4%) were HCV seronegative at baseline, and 540 (52.7%) of them had a second visit, including 376 cohort participants and 164 RDS recaptures. Participants had a median age of 40 years (IQR: 33–47), 93% were male, 75% had education from middle school, and 95.7% had a regular place to stay (Table 1). About 13.5% of them were living with HIV, and 64.6% of PLWH were currently receiving ART. Almost one-fifth of participants had been injecting heroin for more than 15 years, and 33.9% were using methadone at baseline. Participants who had no follow-up visits were younger, more likely to be male and be on methadone, and less likely to be long-term injectors or PLWH than those who had at least one follow-up visit (Table 1).

**Table 1:** Baseline characteristics of hepatitis C-seronegative people who inject drugs participants of the primary study in Hai Phong, Vietnam, stratified by their follow-up status

Characteristics	At least 1 follow-up visit (*N* = 540)—*n* (%)	No follow-up visit (*N* = 485)—*n* (%)	*p* value^h^
Sex: male	502 (93.0)	469 (97.3)^d^	0.001
Median age [IQR]	40 [33–47]	35 [29–44]^b^	< 0.001
Had education from middle school	405 (75.0)	348 (72.1)^b^	0.285
Had a regular place to stay	517 (95.7)	460 (95.2)^b^	0.699
Recent monthly income: > 3 million VND^g^	462 (85.6)	436 (90.3)^b^	0.022
Lived with a sexual partner	230 (42.6)	217 (44.9)^b^	0.452
HIV positive	73 (13.5)	13 (2.7)^e^	< 0.001
Currently on ART (self-reported)	42 (64.6)^f^	6 (50.0)^a^	0.861
Years of heroin injection^c^			< 0.001
< 5 years	197 (36.5)	215 (44.5)	
5 to < 10 years	138 (25.6)	140 (29.0)	
10 to < 15 years	103 (19.1)	79 (16.4)	
≥ 15 years	102 (18.9)	49 (10.1)	
Currently using methadone at baseline	183 (33.9)^a^	256 (52.8)^d^	0.017

^a^1 missing value

^b^2 missing values

^c^2 missing values in the group of no follow-up

^d^3 missing values, visit

^e^4 missing values

^f^8 missing values

^g^3 million VND ≈ 130 USD

^h^Based on Chi square tests for categorical variables and Wilcoxon Rank Sum test for continuous variables

### Estimation of HCV incidence

**Fig. 1:**
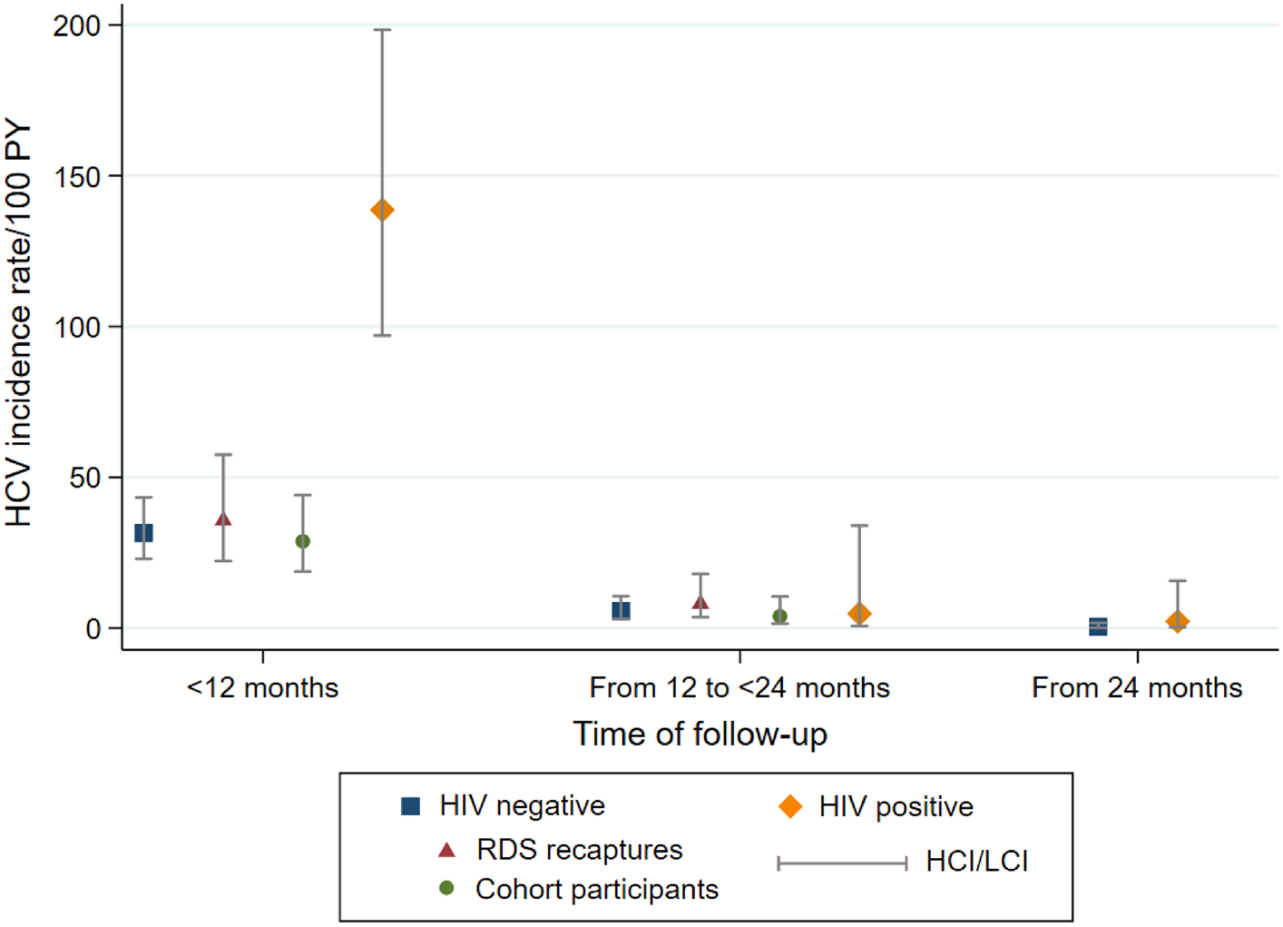
Hepatitis C incidence trend per follow-up time of participants. Generated using STATA /IC 16.1

Participants were followed for 844 person-years, including 242 person-years for RDS recaptures and 602 person-years for cohort participants. Overall, 83 participants seroconverted for HCV, yielding an incidence rate of 9.8/100 person-years (95% CI 7.9–12.2). Among cohort participants, 58 seroconverted for an incidence of 9.6/100 person-years (95% CI 7.5–12.5), while among RDS recaptures, 25 participants seroconverted for an incidence of 10.3 (95% CI 7.0–15.3). HCV incidence was highest among participants aged 30–39 years (14.3/100 person-years) (Table 2). Furthermore, HCV incidence was 5.6 times (*p* < 0.001) higher among PLWH than their counterparts, regardless of age group. Among HIV-negative participants, HCV incidence was almost double for RDS recaptures compared to cohort participants (10.0 and 5.1 per 100 person-years, *p* = 0.01). Most HCV-negative participants (83%) seroconverted during the first year of follow-up, resulting in the highest HCV incidence rate (Fig. 1). As a result, the difference was distinctive between subgroups in this period with the incidence rates higher for PLWH and RDS recaptures compared to their counterparts, i.e., HIV-negative and cohort participants, respectively.

**Table 2:** Hepatitis C incidence by age groups, HIV and cohort participation status

	No of cases	Person-years	Incidence rate (per 100 person-years)		95% CI	Incidence rate ratio^b^	*p*-value
HCV incidence by age groups							
18–29 years old	8	114.4		7.0	3.5–14.0	1	
30–39 years old	39	272.8		14.3	10.5–19.6	2.0	0.060
40–54 years old	30	356.4		8.4	5.9–12.0	1.2	0.641
55–72 years old	6	100.3		6.0	2.7–13.3	0.9	0.772
HCV incidence by HIV and cohort participation status^a^							
HIV negative	50	755.1		6.6	5.0–8.7	1	
RDS recaptures	24	240.1		10.0	6.7–14.9	1	
Cohort participants	26	515.0		5.1	3.4–7.4	0.5	0.014
HIV positive	33	88.7		37.2	26.4–52.3	5.6	< 0.001

^a^As most HIV infected were cohort participants, stratification by cohort status was done only for HIV-negative group

^b^Rate ratios were calculated with the Mantel–Haenszel method

### Risk factor analysis

#### Univariable analysis

Overall, 245 controls were randomly selected for 83 cases from participants who remained HCV seronegative and met matching criteria. Most cases had 3 controls, but 2 cases had only one control. There was only 1 case in each stratified group who shared drugs with used syringes, helped someone injecting for the first time, injected with a used needle/syringe, had tattoo/piercing or experienced an overdose; therefore, these variables were excluded from further analysis. Most participants were male, and slightly older in cohort group than in RDS-recaptured participants (Table 3). As all participants with multiple RDS participations currently injected heroin, active heroin injection could only be assessed among cohort participants, with 81.0% and 65.1% were still injecting heroin in the HCV-positive and -negative groups, respectively.

**Table 3:** Characteristics of participants who were hepatitis C-seronegative at baseline, stratified by cohort participation and hepatitis C-seroconversion status

Characteristics	Categories	RDS recaptures (*N* = 98)		Cohort participants (*N* = 230)	
		HCV seroconversion status		HCV seroconversion status	
		Yes (*N* = 25) *n* (%)	No (*N* = 73) *n* (%)	Yes (*N* = 58) *n* (%)	No (*N* = 172) *n* (%)
Sex^a^	Male	25 (100.0)	73 (100.0)	57 (98.3)	171 (99.4)
Median age [IQR]^a^		34 [31–45]	35 [30–43]	39.5 [35–45]	42 [34–47]
Had education from middle school		20 (80.0)	59 (80.8)	34 (58.6)	134 (77.9)
Had a regular place to stay^a^		23 (92.0)	68 (93.2)	57 (98.3)	165 (95.9)
Recent monthly income^a,c^	≥ 3 million VND	20 (80.0)	62 (84.9)	53 (91.4)	141 (82.0)
Lived with a sexual partner^a^		17 (68.0)	27 (37.0)	19 (32.8)	69 (40.1)
HIV positive^a^		1 (4.0)	1 (1.4)	32 (55.2)	22 (12.80)
Currently on ART among HIV positive^e^		0	0	17 (63.0)	13 (61.9)
Years of heroin injecting^a^					
	< 5 years	12 (48.0)	28 (38.4)	11 (19.0)	62 (36.1)
	5 to < 10 years	6 (24.0)	24 (32.9)	15 (25.9)	45 (26.2)
	10 to < 15 years	2 (8.0)	12 (16.4)	12 (20.7)	32 (18.6)
	≥ 15 years	5 (20.0)	9 (12.3)	20 (34.5)	33 (19.2)
Active heroin injection^b^		25 (100.0)	73 (100.0)	47 (81.0)	112 (65.1)
Daily injection^b^		17 (68.0)	50 (68.5)	25 (43.1)	52 (30.2)
Methamphetamine smoking^b^		10 (40.0)	34 (46.6)	22 (37.9)	67 (39.0)
Use of non-injected drugs^b,f^		4 (20.0)	8 (14.3)	5 (8.8)	15 (8.9)
Had sex while taking methamphetamine^b^		3 (12.2)	9 (12.3)	6 (10.3)	25 (14.5)
Incarcerated overnight^b^		3 (12.0)	5 (6.9)	2 (3.5)	6 (3.5)
Currently using methadone^b^		14 (56.0)	59 (80.8)	37 (63.8)	93 (54.1)
At-risk consumption of alcohol^b,d^		3 (12.0)	20 (27.4)	14 (24.1)	60 (34.9)
Use of water/novocain used by others^b,g^		5 (20.0)	9 (12.3)	2 (3.5)	7 (4.2)

^a^Data taken at the inclusion visit

^a^Data taken at the index visit

^c^3 million VND ≈ 130 USD

^d^An AUDIT-C score of ≥ 4 for male or ≥ 3 for female was considered as at-risk alcohol consumption

Missing values for HCV seroconverters and HCV non-seroconverters respectively:

^e^5 and 1 among cohort participants; 1 and 0 among RDS recaptures

^f^1 and 4 among cohort participants; 5 and 17 for RDS recaptures

^g^1 and 4 among cohort participants

Among RDS-recaptured participants, age, living with a sexual partner, currently using methadone, and at-risk consumption of alcohol tended to be associated with HCV seroconversion in univariable analyses (Table 4). For cohort participants, PLWH, still injecting heroin, age, education, recent monthly income, daily injection, currently using methadone, and at-risk alcohol consumption were likely associated with HCV seroconversion.

**Table 4:** Factors associated with hepatitis C seroconversion from univariable analysis using conditional logistic regression, stratified by cohort participation status

Variables	RDS recaptures		Cohort participants	
	Crude OR (95% CI)	*p* value	Crude OR (95% CI)	*p* value
Age	0.9 (08–1.2)	0.506	0.9 (0.8–1.0)	0.024
Had education from middle school	0.9 (0.3–2.9)	0.882	0.4 (0.2–0.8)	0.008
Had a regular place to stay	0.8 (0.1–4.6)	0.819	2.3 (0.3–19.0)	0.428
Recent monthly income (≥ 3 million VND)^a^	0.7 (0.2–2.5)	0.632	2.5 (0.9–7.4)	0.095
Lived with a sexual partner	3.7 (1.3–10.7)	0.014	0.7 (0.3–1.3)	0.265
HIV positive			7.9 (3.7–16.8)	< 0.001
Active heroin injection			2.4 (1.1–5.1)	0.020
Daily injection	0.9 (0.4–2.5)	0.901	1.8 (0.9–3.4)	0.080
Methamphetamine smoking	0.8 (0.3–1.9)	0.564	1.0 (0.5–1.8)	0.874
Use of non-injected drugs	1.4 (0.4–5.2)	0.603	1.0 (0.3–3.0)	1.000
Had sex while taking methamphetamine	1.0 (0.3–4.0)	1.000	0.7 (0.3–1.8)	0.439
Incarcerated overnight	1.9 (0.4–8.8)	0.401	1.0 (0.2–5.0)	1.000
Currently using methadone	0.3 (0.1–0.9)	0.028	1.6 (0.8–2.9)	0.170
At-risk consumption of alcohol	0.4 (0.1–1.3)	0.121	0.6 (0.3–1.2)	0.130
Use of water/novocain used by others	2.0 (0.5–7.4)	0.296	0.7 (0.1–3.7)	0.696

^a^3 million VND ≈ 130

#### Multivariable analysis

For RDS-recaptured participants, after 10 iterations of random selection of controls and multivariable analyses using the best-fit approach, age, living with a sexual partner, and currently using methadone were retained in the final model. From the bootstrapping program, RDS recaptures who were legally married/lived with partners had 2.9 times (95% CI 2.2–4.3) higher odds of seroconverting for HCV than those not living with partners. On the other hand, people who were currently using methadone had much lower odds of contracting HCV, i.e., OR: 0.4, 95% CI (0.3–0.5) (Table 5). Following the same approach, age, HIV status, and active heroin injection were retained in the final model for cohort participants. The bootstrapped odd ratios were 10.0 (95% CI 6.8–16.1) for HIV infection and were 3.2 (95% CI 2.3–4.8) for active heroin injection (Table 5).

**Table 5:** Factors associated with hepatitis C seroconversion from multivariable analysis using conditional logistic regression and bootstrapped program of 1000 iterations, stratified by cohort participation status

Variables	RDS recaptures	Cohort participants
	Bootstrapped adjusted OR (95% CI)	Bootstrapped adjusted OR (95% CI)
Age	0.9 (0.8–1.0)	0.9 (0.8–1.0)
Lived with a sexual partner	2.9 (2.2–4.3)	NS
HIV positive	NA	10.0 (6.8–16.2)
Active heroin injection	NA	3.2 (2.3–4.8)
Currently using methadone	0.4 (0.3–0.5)	NS

NA not appropriate (no PLWH among RDS recaptures; all RDS recaptures currently injected heroin), NS not included/retained in the final model (no association in univariable/multivariable analysis)

## Discussion

Overall, the HCV incidence rate was still high among PWID in Hai Phong, Vietnam, especially among PLWH. HCV incidence tended to decrease over follow-up time and be lower among cohort participants. PLWH, living with a sexual partner, and active heroin injection were associated with a higher risk of HCV seroconversion, while currently using methadone seemed to reduce this risk.

The overall HCV seroconversion rate was close to 10/100 person-years, almost 5 times higher than the WHO elimination target of HCV incidence for PWID [3], with a higher rate among those aged 30–39 years old (14.3/100 person-years). This shows the high dynamic of HCV transmission and the difficulty in achieving the elimination target among PWID in Hai Phong in the absence of an HCV treatment program. However, this result is half of what was estimated in the DRIVE-IN study 2 years before the beginning of this work [6]. There was also a substantial overall reduction in HCV incidence with time, and a lower incidence among cohort participants after being stratified by HIV infection. Even though participants with 1 year or more of follow-up were different from those having less than 1 year of follow-up (data not shown), this difference does not help to explain the above-mentioned reduction in HCV incidence. Alternatively, these results suggest an impact of the DRIVE project on HCV prevention through the HCV harm reduction counseling as participants with longer follow-up or in cohort benefited from more counseling sessions. Indeed, one-third of cohort participants stopped injecting during the follow-up (data not shown), which lowered considerably their risk of HCV infection. However, the cohort effect could not be excluded as most HCV seroconversion occurred within the first year of follow-up, representing a very high initial risk of HCV infection in the study population.

Unexpectedly, we found that the HCV incidence rate was much higher among participants living with HIV (OR = 10.0), even after adjusting for active heroin injection. Only two studies conducted among drug users showed some evidence of association between HIV infection and HCV incidence, i.e., HR = 2.1 (95% CI 1.4–3.6) in China and HR = 1.8 (95% CI 1.0–3.1) in Canada [12, 13]. The strong association between HIV status and HCV seroconversion found in our study is unlikely to be confounded by risky behaviors as PLWH declared injecting less frequently, with less risky injecting practices than HIV-negative PWID (Supplementary Table 1). Although risky behaviors could still be underreported by PLWH, their very high odds of HCV seroconversion may also be related to some characteristics of chronic HIV infection, which make them more susceptible to HCV infection, such as residual immune activation or immune exhaustion even among well-treated PLWH. The association between these abnormal immune reactions with HCV co-infection was highlighted in a Norwegian study [14].

The main route of HCV acquisition among PWID is well known through drug injection. The OR of heroin injection and HCV incidence estimated in this study is similar to that of an Italian study, i.e., 2.6 (95% CI 1.0–6.7) [15]. However, known mechanisms of HCV transmission, such as sharing water/novocaine, sharing needles/syringes or having new tattoo/piercing [16, 17], were not associated with HCV seroconversion in our study. This may be due to the low frequency (or underreporting) of these unsafe behaviors in the study population, which agrees with previous studies in Haiphong [6, 18].

Living with a sexual partner was also associated with an increased risk of HCV seroconversion (OR = 2.9), but only among participants recaptured in RDS surveys. However, most of these participants reported having no partners who had ever injected (data not shown). The association between living with a sexual partner and HCV prevalence, but not HCV incidence, has been inconsistently reported in some papers [19, 20]. As expected, among RDS recaptures, currently using methadone was a protective factor against HCV seroconversion (OR = 0.4), as previously reported [21, 22]. While all RDS-recaptured participants were active PWID, almost one-third of cohort participants had stopped injecting during follow-up, which may explain the absence of a similar protective effect among cohort participants.

This study has several limitations. The risk factor analysis could be subject to residual confounding as more complete adjustment could not be made due to the low reported frequency of some known risk factors. The low presence of women in the study participants, reflecting the study population characteristics, reduces the generalization of study results to this subgroup. Selection bias may be less of an issue in this study as those who had at least two visits were not very different from those who participated only once. Data on risky behaviors were self-reported by participants, which are prone to recall bias and desirability bias, despite the involvement of peer educators to reduce the latter.

The DRIVE model was more effective in reducing HIV infection than HCV infection although these infections have similar routes of transmission [6]. This may be due to high prevalence of untreated hepatitis C (60.6%) among PWID in Hai Phong [23], which may stochastically increase the number of events with high risk of transmission in the population. Therefore, to further decrease incidence in Hai Phong and similar settings, the availability of free or affordable HCV treatments for PWID to reduce force of infection, is urgently needed. Our group showed that such intervention, using existing health services with involvement of CBO, can lead to high HCV cure rates in this community [23]. In addition, easier access to methadone programs and more intensive CBO-led harm reduction activities should be implemented. Apart from providing clean syringes and needles, these activities need to include information sessions about the known risk of HCV transmission through unsafe injecting practices such as water sharing or frontloading. The very strong association between HIV infection and seroconversion calls for prioritizing PWID living with HIV for these activities. In addition, routine and frequent periodic HCV screening should be recommended for PWID, especially in HIV and methadone clinics, to detect new HCV cases. Finally, more studies should investigate the relationship between HIV-positive status (i.e., residual immune dysfunction) and HCV incidence.

## Conclusion

HCV incidence was still high among PWID in 2020 in Hai Phong, Vietnam, with a much higher risk among PLWH, after adjustment for confounders. HCV elimination target in PWID cannot be achieved without large-scale free HCV treatment alongside effective HCV prevention interventions targeting PWID.

## Supplementary Information

Below is the link to the electronic supplementary material.

Supplementary file1 (DOCX 507 KB)
