# Supplementary material for: Hepatitis C virus incidence trend and its risk factors among people who inject drugs in Hai Phong, Vietnam
Source: Hepatol Int. 2025 Jul 17;19(5):1077–86. doi: 10.1007/s12072-025-10856-w (PMC12508010; doi:10.1007/s12072-025-10856-w)
Supplement: Supplementary file 1 — Supplementary file1 (DOCX 507 KB) [file 12072_2025_10856_MOESM1_ESM.docx]

**SUPPLEMENTARY DOCUMENTS**

**Supplementary figure 1:** Description of the four RDS surveys and establishment of the cohorts: 200-400 participants were selected from participants at each RDS to form HIV negative and HIV positive cohorts (from the DRIVE protocol)


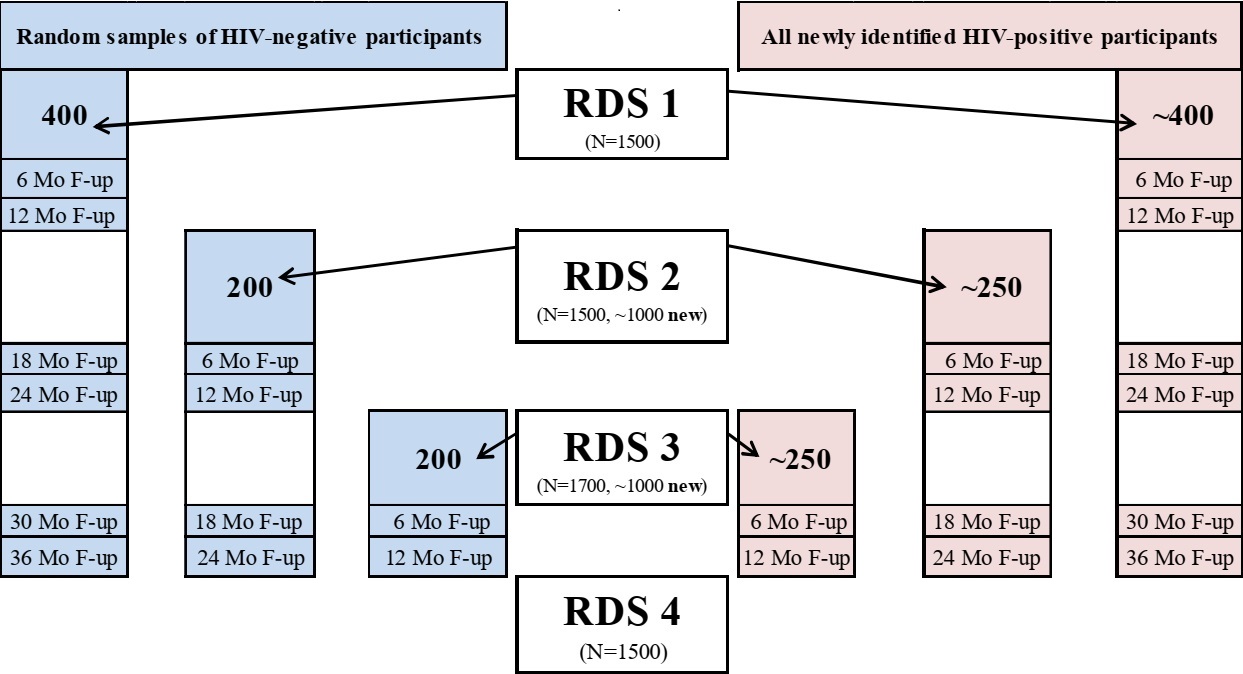


10/2019-01/2020

**HIV negative cohort**

**HIV positive cohort**

10/2016-01/2017

10/2018-01/2019

10/2017-01/2018

**Supplementary table 1:** Distribution of drug use and risky injecting practice variables among PLWH and participants without HIV

| ***Characteristics*** | ***HIV positivity* (N=56)**  n (%) | ***HIV negativity* (N=272)**  n (%) |
| --- | --- | --- |
| **Active heroin injection** | *39 (69.6)* | *218 (81.2)* |
| **Daily injection** | *20 (35.7)* | *124 (45.6)* |
| **Methamphetamine smoking** | *18 (32.1)* | *115 (42.3)* |
| **Use of non-injected drugs^a^** | *5 (9.1)* | *27 (11.0)* |
| **Having sex while taking methamphetamine** | *5 (8.9)* | *38 (14.0)* |
| **Incarcerated overnight** | *2 (3.6)* | *14 (5.2)* |
| **At-risk consumption of alcohol** | *14 (25.0)* | *83 (30.5)* |
| **Use of water/novocain used by others** | *2 (1.8)* | *22 (8.1)* |
| **Injecting with used syringes/needles** | *0* | *7 (2.6)* |
| **Sharing drugs with used syringes^b^** | *0* | *8 (3.0)* |
| **Having new tattoo/piercings^c^** | *1 (1.8)* | *4 (1.5)* |

**^a^***1 missing value for PLWH; 26 missing values for participants without HIV*

**^b^***2 missing values for PLWH; 3 missing values for participants without HIV*

**^c^***3 missing values for participants without HIV*
